# Coffee consumption and cancer risk in African Americans from the Southern Community Cohort Study

**DOI:** 10.1038/s41598-020-72993-6

**Published:** 2020-10-21

**Authors:** Stephanie L. Schmit, Onyekachi Nwogu, Marco Matejcic, Amanda DeRenzis, Loren Lipworth, William J. Blot, Leon Raskin

**Affiliations:** 1 Department of Cancer Epidemiology, H. Lee Moffitt Cancer Center and Research Institute, Tampa, FL USA; 2 Department of Gastrointestinal Oncology, H. Lee Moffitt Cancer Center and Research Institute, Tampa, FL USA; 3 Division of Epidemiology, Department of Medicine, Vanderbilt University Medical Center, Nashville, TN USA; 4 Vanderbilt Center for Translational and Clinical Cardiovascular Research, Vanderbilt University Medical Center, Nashville, TN USA; 5 The International Epidemiology Institute, Rockville, MD USA

**Keywords:** Cancer epidemiology, Cancer prevention, Statistical methods, Risk factors

## Abstract

Coffee consumption has been associated with the risk of cancer at several anatomical sites, but the findings, mostly from studies of non-Hispanic whites and Asians, are inconsistent. The association between coffee consumption and the incidence of cancer has not been thoroughly examined in African Americans. We conducted a nested case–control study including 1801 cancer cases and 3337 controls among African Americans from the Southern Community Cohort Study (SCCS) to examine the association between coffee drinking, as assessed by a semi-quantitative food frequency questionnaire, and the risk of four common cancers (lung, prostate, breast, colorectal). We used logistic regression adjusted for age, sex and cancer-specific risk factors. Overall, only ≤ 9.5% of African American cases and controls from the SCCS drank regular or decaffeinated coffee ≥ 2 times/day. After adjustment for major cancer-specific risk factors, coffee consumption was not statistically significantly associated with the risk of lung, breast, colorectal, or prostate cancers (OR range 0.78–1.10; *P* ≥ 0.27 for ≥ 2 versus < 1 times/day) or overall cancer risk (OR 0.93; 95% CI 0.75–1.16; *P* = 0.52 for ≥ 2 versus < 1 times/day). Coffee consumption was not associated with the risk of cancer among African Americans in our study.

## Introduction

Coffee is one of the most commonly consumed beverages in the United States^1^. It consists of hundreds of compounds including caffeine, melanoidins, diterpenes, polyphenols, trigonelline, lipids, and complex polysaccharides, each with a wide range of effects on normal physiology and pathophysiology of human diseases^2,3^. Several natural compounds found in coffee exert inhibitory effects on cancer initiation and development^4,5^. For example, caffeine is a well-characterized psychostimulator and diuretic that decreases cancer cell proliferation^6^, while polyphenols exert antioxidant activity (chlorogenic and caffeic acids)^7,8^ and inhibit DNA strand breaks (ferulic acid)^9^.

Cancer accounts for a major burden of disease in the United States, with over 1.7 million new cases and 600,000 deaths estimated in 2019^10^. There are clear disparities in cancer incidence and mortality rates across racial/ethnic groups^11^. Incidence rates of lung and breast cancer are highest among non-Hispanic whites, with ~ 40% and ~ 110% higher rates compared to Hispanics, respectively. Non-Hispanic Blacks have ~ 50% higher incidence rates of colorectal cancer and > 200% higher incidence rates of prostate cancer as compared to the lowest rates in Asians/Pacific Islanders. The mortality rates for non-Hispanic Blacks are also the highest among all racial/ethnic groups, with percent increases ranging from ~ 98% for lung cancer to ~ 350% for prostate cancer compared to Asians/Pacific Islanders. These racial/ethnic disparities in incidence and mortality rates are not fully understood, but may be due, in part, to differences in environmental exposures and nutritional habits^12^.

Epidemiologic studies have reported an inverse association between coffee consumption and many cancer types, which is consistent with the anti-carcinogenic activity of coffee components^13^. A protective association for coffee has been demonstrated in several cancers including colorectal, prostate, liver, endometrial, and oral/pharyngeal^14–18^. However, coffee consumption was found to be associated with elevated risk of lung cancer, although the association was substantially attenuated after adjustment for smoking^19,20^. The association between coffee consumption and cancer may differ across racial/ethnic groups with different consumption patterns and distributions of other risk factors. To date, most epidemiological studies of coffee consumption and cancer risk have been conducted in non-Hispanic White or Asian populations. To our knowledge, few studies have investigated this relationship in African Americans, the racial group that suffers the greatest burden of many cancers^11^. A prospective study in the Multiethnic Cohort reported a reduced risk of overall cancer associated with high coffee consumption in African Americans after adjustment for smoking and other potential confounders^21^, although the inverse association was not statistically significant for individual cancer sites^21,22^. Similarly, the Black Women’s Health Study found no association between coffee consumption and the risk of breast cancer in African American women^23^.

We designed a nested case–control study within the Southern Community Cohort Study (SCCS) to investigate the association between coffee consumption and the risk of the most prevalent cancers in the US (lung, prostate, breast and colorectal) in African Americans. The SCCS is a large prospective cohort comprised largely of medically underserved participants in the southeastern United States, with nearly 70% of study participants self-reporting as African American^24^.

## Results

### Characteristics of the study participants

Demographic and exposure characteristics of participants analyzed in this study are summarized and compared between cases and controls in Table 1. Cases included African American participants with one of the major cancer types: lung cancer (n = 524), prostate cancer (n = 503), breast cancer (n = 458), and colorectal cancer (n = 316) (Table 1). Controls were 3,337 African American participants without cancer. Characteristics of study participants summarized by categories of total coffee consumption are presented in Supplementary Table S1.

**Table 1:** Characterization of Southern Community Cohort Study African American cancer cases and controls included in the coffee consumption analysis.

	Controls (n = 3,337)	Lung and bronchus cancer cases (n = 524)	p-value^1^	Prostate cancer cases (n = 503)^2^	p-value	Breast cancer cases (n = 458)^3^	p-value	Colorectal cancer cases (n = 316)	p-value
Age [mean (sd)]	55.6 (8.9)	55.3 (8.6)	0.454	57.9 (7.9)	9.6 × 10^–7^	54.1 (8.8)	0.015	55.4 (8.7)	0.689
**Sex [n (%)]**			1.7 × 10^–5^		–		–		0.007
Male	1633 (48.9)	310 (59.2)		503 (1)		0 (0)		129 (40.8)	
Female	1704 (51.1)	214 (40.8)		0 (0)		458 (1)		187 (59.2)	
**Education [n (%)]**			2.0 × 10^–5^		0.011		0.083		0.755
< 12 years	1240 (37.2)	244 (46.6)		180 (35.8)		132 (28.8)		111 (35.1)	
Graduated high school	1005 (30.1)	155 (29.6)		145 (28.8)		143 (31.2)		104 (32.9)	
Some college	754 (22.6)	97 (18.5)		103 (20.5)		124 (27.1)		68 (21.5)	
Graduated college or higher	335 (10.0)	28 (5.3)		75 (14.9)		59 (12.9)		32 (10.1)	
Unknown^4^	3 (0.1)	0 (0)		0 (0)		0 (0)		1 (0.3)	
**Income, US dollar [n (%)]**			7.8 × 10^–6^		1.2 × 10^–4^		0.578		0.810
< 15,000	1993 (59.7)	366 (69.8)		252 (50.1)		265 (57.9)		191 (60.4)	
15,000—24,999	691 (20.7)	104 (19.8)		113 (22.5)		104 (22.7)		60 (19.0)	
24,999—49,999	427 (12.8)	41 (7.8)		81 (16.1)		57 (12.4)		45 (14.2)	
≥ 50,000	178 (5.3)	11 (2.1)		52 (10.3)		26 (5.7)		16 (5.1)	
Unknown	48 (1.4)	2 (0.4)		5 (1.0)		6 (1.3)		4 (1.3)	
**BMI [mean (sd)]**	30.2 (7.3)	26.4 (6.1)	< 2.2 × 10^–16^	28.3 (5.8)	0.261	33.2 (7.8)	0.040	30.3 (6.9)	0.911
**Total energy intake, kcal/day [mean (sd)]**	2624.9 (1502.3)	2978.9 (1665.9)	3.70 × 10^–06^	2842.6 (1432.7)	0.230	2267.9 (1277.3)	0.876	2584.1 (1538.2)	0.366
**Total activity MET-hours [mean (sd)]**	21.5 (19.1)	21.1 (19.0)	0.668	20.6 (19.1)	0.014	20.4 (16.3)	0.621	20.3 (17.0)	0.223
**Smoking [n (%)]**			< 2.2 × 10^–16^		0.004		0.987		0.035
Never	1207 (36.2)	30 (5.7)		136 (27.0)		228 (49.8)		123 (38.9)	
Former	795 (23.8)	96 (18.3)		153 (30.4)		97 (21.2)		89 (28.2)	
Current	1317 (39.5)	396 (75.6)		211 (41.9)		131 (28.6)		102 (32.3)	
Unknown	18 (0.5)	2 (0.4)		3 (0.6)		2 (0.4)		2 (0.6)	
**HRT [n (%)]**^**5,6**^			0.377		–		0.359		0.940
Never (premenopause)	416 (24.4)	43 (20.1)		–		120 (26.2)		47 (25.1)	
Ever (premenopause)	22 (1.3)	1 (0.5)		–		10 (2.2)		3 (1.6)	
Never (postmenopause)	838 (49.2)	113 (52.8)		–		210 (45.9)		88 (47.1)	
Ever (postmenopause)	420 (24.6)	55 (25.7)		–		116 (25.3)		48 (25.7)	
Unknown	8 (0.5)	2 (0.9)		–		2 (0.4)		1 (0.5)	
**Vegetable consumption, times/day [n (%)]**			0.260		0.176		0.848		1
< 5	3252 (97.5)	514 (98.1)		498 (99.0)		442 (96.5)		308 (97.5)	
≥ 5	58 (1.7)	5 (1.0)		4 (0.8)		9 (2.0)		6 (1.9)	
Unknown	27 (0.8)	5 (1.0)		1 (0.2)		7 (1.5)		1 (0.6)	
**Current high cholesterol medication use [n (%)]**^**7**^			0.685		0.477		0.543		0.962
No	455 (13.6)	61 (11.6)		64 (12.7)		81 (17.7)		42 (13.3)	
Yes	661 (19.8)	81 (15.4)		110 (21.9)		103 (22.5)		59 (18.7)	
Unknown	2 (0)	1 (0.2)		0 (0)		0 (0)		0 (0)	
**Daily low-dose aspirin use, years [n (%)]**			0.162		0.074		0.640		1
< 2	2833 (84.9)	460 (87.8)		413 (82.1)		382 (83.4)		271 (85.8)	
≥ 2	430 (12.9)	56 (10.7)		78 (15.5)		66 (14.4)		41 (13.0)	
Unknown	74 (2.2)	8 (1.5)		12 (2.4)		10 (2.2)		4 (1.3)	
**NSAID use, years [n (%)]**^**8**^			0.345		0.540		1		0.503
< 1	2549 (76.4)	408 (77.9)		400 (79.5)		340 (74.2)		238 (75.3)	
≥ 1	725 (21.7)	103 (19.7)		90 (17.9)		110 (24.0)		75 (23.7)	
Unknown	63 (1.9)	13 (2.5)		13 (2.6)		8 (1.7)		3 (0.9)	
**Alcohol consumption, times/day [n (%)]**^**9**^			2.35 × 10^–11^		0.061		0.859		0.015
< 1	2556 (76.6)	338 (64.5)		355 (70.6)		400 (87.3)		260 (82.3)	
≥ 1 and < 2	195 (5.8)	29 (5.5)		36 (7.2)		16 (3.5)		20 (6.3)	
≥ 2	532 (15.9)	149 (28.4)		104 (20.7)		34 (7.4)		31 (9.8)	
Unknown	54 (1.6)	8 (1.5)		8 (1.6)		8 (1.7)		5 (1.6)	
**Sweet beverage consumption, times/day [n (%)]**^**10**^			0.046		0.021		0.632		0.158
< 1	997 (29.9)	132 (25.2)		168 (33.4)		150 (32.8)		79 (25.0)	
≥ 1 and < 2	832 (24.9)	125 (23.9)		133 (26.4)		120 (26.2)		81 (25.6)	
≥ 2	1476 (44.2)	258 (49.2)		201 (40.0)		180 (39.3)		154 (48.7)	
Unknown	32 (1.0)	9 (1.7)		1 (0.2)		8 (1.7)		2 (0.6)	
**Daily tea intake, times/day [n (%)]**			0.074		0.384		0.529		0.142
< 1	2960 (88.7)	448 (85.5)		451 (89.7)		400 (87.3)		276 (87.3)	
≥ 1 and < 2	228 (6.8)	50 (9.5)		35 (7.0)		38 (8.3)		30 (9.5)	
≥ 2	108 (3.2)	18 (3.4)		12 (2.4)		11 (2.4)		7 (2.2)	
Unknown	41 (1.2)	8 (1.5)		5 (1.0)		9 (2.0)		3 (0.9)	
**Daily regular coffee intake, times/day [n (%)]**			1.4 × 10^–4^		0.891		0.052		0.998
< 1	2456 (73.6)	337 (64.3)		360 (71.6)		346 (75.5)		231 (73.1)	
≥ 1 and < 2	614 (18.4)	127 (24.2)		104 (20.7)		82 (17.9)		59 (18.7)	
≥ 2	232 (7.0)	50 (9.5)		32 (6.4)		18 (3.9)		22 (7.0)	
Unknown	35 (1.0)	10 (1.9)		7 (1.4)		12 (2.6)		4 (1.3)	
**Daily decaffeinated coffee intake, times/day [n (%)]**			0.033		0.330		0.994		0.881
< 1	2982 (89.4)	445 (84.9)		457 (90.9)		403 (88.0)		285 (90.2)	
≥ 1 and < 2	238 (7.1)	52 (9.9)		28 (5.6)		32 (7.0)		23 (7.3)	
≥ 2	71 (2.1)	15 (2.9)		12 (2.4)		11 (2.4)		5 (1.6)	
Unknown	46 (1.4)	12 (2.3)		6 (1.2)		12 (2.6)		3 (0.9)	

^1^Statistical significance for differences between cases and controls was tested using t-test for continuous variables and chi-square test for categorical variables.

^2^Comparisons are to male controls only.

^3^Comparisons are to female controls only.

^4^Unknown subjects were excluded from calculations.

^5^HRT = hormone replacement therapy.

^6^In females only.

^7^Among those self-reporting high cholesterol.

^8^NSAID (nonsteroidal anti-inflammatory drugs) include any of the following drug classes: Aspirin, OTC, Rx; subjects with unknown information for all classes were recoded as unknown in the NSAID use variable.

^9^Including beer, wine and liquor.

^10^Including soft drinks, Kool-Aid or other sweetened drinks.

### Coffee consumption and risk of cancer

Lung cancer cases consumed more regular (*P* = 1.4 × 10^–4^) and decaffeinated (*P* = 0.033) coffee than controls; 33.7% of lung cancer cases consumed regular coffee ≥ 1 times/day as compared to controls (25.4%) and other cancer cases (from 21.8% to 27.1%) (Table 1). Similarly, 12.8% of lung cancer patients consumed decaffeinated coffee one or more times per day as compared to 9.2% of controls and 8.0%-9.4% for cases of other cancer sites. No statistically significant difference in coffee consumption was observed for other cancer cases compared to controls.

The association between coffee consumption and risk of cancer in African Americans was estimated for lung, prostate, breast, and colorectal cancers separately and combined. There are two multivariable models presented in Table 2: (1) adjusted for the case–control frequency matching factors only (age and sex) and (2) fully adjusted for the covariates relevant to each specific cancer type. In the minimally adjusted model, consumption of regular or decaffeinated coffee was statistically significantly associated with increased risk of lung cancer (*P* = 3.3 × 10^–4^ for 1–2 times/day and 4.1 × 10^–4^ for ≥ 2 times/day). However, this association was no longer statistically significant in the fully-adjusted model including BMI and smoking (*P* = 0.063 for 1–2 times/day and 0.726 for ≥ 2 times/day). No statistically significant associations between coffee consumption and cancer risk were observed for prostate, breast, colorectal, or all four cancers combined. The fully-adjusted ORs for the associations with ≥ 2 versus < 1 times/day were all below 1.0, indicating a tendency towards a reduced cancer risk with higher coffee consumption. Acknowledging our limited statistical power, similar results were observed when each type of coffee was considered separately (Supplementary Tables S2 and S3) or when the reference category included only non-drinkers (data not shown).

**Table 2:** Association between total coffee consumption and cancer risk in Southern Community Cohort Study African Americans.

Coffee intake (times/day)	n (cases/controls)	Adjusted for age and sex^a^		Fully adjusted^b^	
		OR (95% CI)	p-value^c^	OR (95% CI)	p-value
**All cancers**					
< 1	1149/2229	1.00 (Ref)		1.00 (Ref)	
≥ 1 and < 2	410/681	1.16 (1.01–1.34)	0.038	1.09 (0.92–1.30)	0.313
≥ 2	204/375	1.05 (0.87–1.26)	0.606	0.93 (0.75–1.16)	0.519
**Lung cancer**					
< 1	296/2229	1.00 (Ref)		1.00 (Ref)	
≥ 1 and < 2	134/681	1.51 (1.20–1.89)	3.3 × 10^–4^	1.28 (0.99–1.65)	0.063
≥ 2	81/375	1.63 (1.24–2.13)	4.1 × 10^–4^	1.06 (0.77–1.45)	0.726
**Prostate cancer**					
< 1	331/1067	1.00 (Ref)		1.00 (Ref)	
≥ 1 and < 2	111/346	0.95 (0.74–1.22)	0.668	0.91 (0.67–1.23)	0.551
≥ 2	54/197	0.84 (0.61–1.17)	0.315	0.81 (0.55–1.18)	0.273
**Breast cancer**					
< 1	311/1162	1.00 (Ref)		1.00 (Ref)	
≥ 1 and < 2	96/335	1.12 (0.86–1.45)	0.405	1.19 (0.83–1.71)	0.344
≥ 2	37/178	0.78 (0.53–1.14)	0.197	0.80 (0.48–1.33)	0.395
**Colorectal cancer**					
< 1	211/2229	1.00 (Ref)		1.00 (Ref)	
≥ 1 and < 2	69/681	1.08 (0.81–1.44)	0.594	0.86 (0.51–1.46)	0.584
≥ 2	32/375	0.92 (0.62–1.35)	0.656	0.78 (0.39–1.53)	0.47

^a^No sex adjustment for prostate and breast cancers.

^b^Adjustment factors for: All cancers: age, sex, BMI, smoking status and pack-years. Lung cancer: age, sex, BMI, smoking status and pack-years, total energy intake. Prostate cancer: age, BMI, smoking status and pack-years, physical activity. Breast cancer: age, BMI, smoking status and pack-years, HRT, physical activity. Colorectal cancer: age, sex, BMI, smoking status and pack-years, physical activity, vegetable consumption, low dose aspirin use, NSAID use.

^c^p-value from the Wald test.

## Discussion

To our knowledge, the present report is among the first to investigate the impact of coffee consumption on the risk of cancer at multiple sites specifically in African Americans. A major strength of this analysis is the size of the SCCS cohort, which enables careful examination of cancer risk factors among African Americans in the southeastern US. We found no association between coffee consumption and risk of cancer overall or by cancer site after adjustment for cancer-specific risk factors.

In prostate, breast, and colorectal cancers, we observed a trend towards a protective association for coffee intake 2 or more times a day, although none of the results were statistically significant. This trend is in line with the recent large case–control and cohort studies that found a protective association between coffee consumption and the risk of cancers of the breast^25^, colon^26^, and prostate^27^ in Whites. In addition, our findings are consistent with a recent case–control study of White British individuals from the UK Biobank cohort^28^.

It has been suggested that coffee exerts its protective effect through a variety of potentially bioactive compounds. As an example, diterpenes such as cafestol and kahweol have been confirmed to exert anticarcinogenic effects by modulating the expression of proteins involved in cellular antioxidant defense or DNA repair^29,30^. Polyphenols such as chlorogenic and caffeic acid are also important constituents of coffee that were reported to inactivate various pathways involved in tumorigenesis (i.e. cell cycle regulation, apoptosis, inflammatory and stress response) through regulation of DNA methyltransferase activity^31^. In addition, chlorogenic-derived constituents have been shown to alter glucose metabolism and insulin sensitivity in rats^32^, which are established markers for some cancer sites in humans^33^.

The Women's Health Initiative Observational Study reported a borderline significant increased risk of colorectal cancer associated with higher coffee consumption, which is in contrast with the potential anticarcinogenic effect of coffee constituents^34^. A lack of association between regular or decaffeinated coffee and colorectal cancer has also reported in different racial/ethnic groups^35^, including African Americans^21^. The species of coffee bean, method of preparation, roasting degree, and serving size all influence the level and potency of bioactive compounds in coffee, and therefore, coffee’s potential health implications^36^. In addition, coffee is a major source of caffeine which has been reported to both stimulate and suppress tumors^37^. These could be potential reasons for differences in association between coffee consumption and cancer risk across studies. Further, the broad geographical distribution of the populations studied (including African Americans) could serve as a source of variability between association analyses due to the presence of region-specific lifestyle or environmental factors that may modify associations between coffee and cancer risk.

Similar to our results in African Americans, a positive association between coffee consumption and lung cancer risk has been reported by several prospective studies^38,39^ including the NIH-AARP Diet and Health Study^20^. However, the association was no longer statistically significant after adjustment for smoking. It is important to note that the NIH-AARP Diet and Health Study was comprised of 90% Whites overall and 96% whites among coffee drinkers of > 2 times a day. Our results are also in line with a previous study in the Multiethnic Cohort based on 964 African American cases with lung cancer^21^. Of note, any positive association between coffee consumption and lung cancer risk may be attributable to either residual confounding by smoking or potential interaction with unknown risk factors, and prospective studies in African Americans with complete exposure assessment are needed to confirm this hypothesis.

Coffee intake can be difficult to measure accurately, as epidemiologic studies typically rely on self-reported data from questionnaires which are hampered by recall bias and measurement error^40^, and most studies (including ours) do not have information on duration of exposure. Further, low consumption of coffee in a population may preclude accurate characterization of a dose–response relationship, as only 7.0% of the African American SCCS controls in our study reported drinking regular coffee ≥ 2 times per day. The same trend was observed for decaffeinated coffee consumption, with only 2.1% of African American SCCS controls drinking coffee ≥ 2 times per day. When considering overall coffee consumption (regular coffee + decaffeinated coffee), 31.6% of healthy African Americans (versus 59.8% of Whites in the SCCS) reported that they were daily coffee drinkers (≥ 1 times/day). Consistent with our observations, it has been reported previously that African Americans drink 2–3 times less coffee than Whites or Mexican Americans regardless of age and sex^41^. This is also in agreement with the low coffee consumption patterns in African countries as compared to European countries^42^. Thus, the low number of coffee drinkers among African Americans suggests the importance of large cohorts for a powerful investigation of coffee’s impact on cancer risk in this population.

In this study, non-drinkers were combined with those who consumed coffee < 1 times/day based on previous studies that reported no significant differences in cancer risk between non-drinkers and very low coffee drinkers in African Americans^21–23^. Having a larger reference group including both non-drinkers and extremely low drinkers increases the statistical power to detect an association between daily coffee consumption and cancer risk. Although previous prospective studies reported significant associations between very high levels of coffee consumption (i.e. ≥ 5 times/day) and cancer risk that were not observed in lower categories of intake^20,25,38,43,44^, the upper end of the administered FFQ limited our ability to investigate the highest levels of coffee consumption in relation to cancer risk. Other study limitations include potential measurement error in dietary coffee consumption due to reporting bias, incomplete food tables and a lack of information on the serving size (e.g. fluid ounces), type, and brewing method of coffee consumed. These factors influence the level of exposure to different bioactive compounds with potential anticarcinogenic effects. Further, the lack of evaluation of other known or unknown confounders (e.g. family history of cancer) that could potentially influence the association between coffee and cancer risk is a limitation.

In conclusion, we confirmed the relatively low consumption of coffee among African Americans in the SCCS and showed that coffee consumption was not strongly associated with risk of any of the four major cancer types in African Americans. Demographic, cultural, environmental, and genetic factors that differ across racial/ethnic groups may modify the association of coffee consumption with cancer risk, and thus, larger cohorts with a higher proportion of coffee drinkers and complete exposure assessment are needed in future studies to further evaluate the relation between coffee consumption and cancer in African Americans.

## Methods

### Study participants and data retrieval from the Southern Community Cohort Study (SCCS)

A detailed description of SCCS methods is available on the study website (https://www.southerncommunitystudy.org/) and in previous publications^24^. Briefly, the SCCS is an ongoing prospective study across 12 southeastern states in the US aiming to investigate chronic diseases in approximately 85,000 adults, two-thirds of whom are African Americans. Recruitment of participants (aged 40–79) began in March 2002 and ended in September 2009. A total of 84,797 participants were enrolled in the SCCS (65% African Americans, 30% White, 5% other racial/ethnic groups). Participants were followed from the date of enrollment until first cancer diagnosis, death, emigration or end of the follow-up period, whichever occurred first. Incident cancer cases were ascertained via linkage to state cancer registries, while deaths were ascertained via linkage to the National Death Index and Social Security mortality files.

At enrollment, participant characteristics including demographic variables, socioeconomic status, medical history, and lifestyle exposures were assessed using a standardized computer-assisted personal interview (available at https://www.southerncommunitystudy.org/). Epidemiological data from the SCCS examined in this study included anthropometric measurements (height, weight, BMI), sociodemographic factors, physical activity, vegetable consumption, smoking, daily alcohol (i.e. beer, wine, liquor), sweet beverage (i.e. soft drinks, Kool-Aid, or other sweetened drinks) and tea consumption, total energy intake, reproductive factors, medication use (e.g. aspirin), and hormone replacement therapy (HRT). Dietary intake, including regular and decaffeinated coffee consumption, was assessed using a validated, semi-quantitative food frequency questionnaire (FFQ) that was developed specifically for this study population^45^. For each food item, the FFQ elicited information on frequency of consumption using the following scale: never, rarely, once a month, 2–3 times a month, once a week, 2–3 times a week, 4–6 times a week, once a day and two or more times a day. Daily regular coffee intake, decaffeinated coffee intake, and total coffee intake (regular coffee + decaffeinated coffee) were each converted into a derived variable including three ordered categories of coffee drinking: < 1 times/day, ≥ 1 and < 2 times/day, and ≥ 2 times/day.

A nested case–control study was designed among recruited participants. Of the 55,362 African American participants recruited to the SCCS, a total of 1,801 cases with incident primary cancers of the lung (N = 524), prostate (N = 503), breast (N = 458), or colorectum (N = 316) were identified. For participants with multiple cancers, we included only the first diagnosed cancer, or if age at diagnosis was the same, we included the cancer occurrence most likely to reflect the primary site based on established metastasis patterns. Controls were randomly selected in a ratio of 5 cancer-free controls per 1 incident lung cancer (the most frequent site in our dataset) case of any racial/ethnic background, employing frequency matching on age at enrollment in 5-year categories, sex, and race (White, Black, other). African Americans among the selected controls (N = 3,337) served as the comparison group in this study. The same control group was used to assess the association with each individual cancer and all cancers combined. Only males (females) among the control group were included in prostate (breast) cancer analyses. The focus on the four major cancers was primarily due to lack of statistical power for analysis of less common cancers.

All methods were performed in accordance with relevant guidelines/regulations. Informed consent was obtained from all participants in the SCCS, and the study was approved by the Institutional Review Boards of Vanderbilt University Medical Center and the Meharry Medical College.

### Statistical analysis

Data are presented as means (± SD) for continuous variables or percentages for categorical variables. We conducted Student’s t-tests and chi-squared tests of independence to compare demographic and risk factor distributions between cases and controls with regard to continuous and categorical variables, respectively. Quantile–quantile (QQ) plots were used to test for deviations from a normal distribution. All analyses were conducted separately by cancer type. Only female controls were included in breast cancer-specific analyses, and only male controls were included in prostate cancer-specific analyses. We estimated the odds ratios (OR) and 95% confidence intervals (CI) for the associations between categories of total coffee consumption and risk of each of the four major cancers (lung, prostate, breast, and colorectal) as well as all four cancers combined using unconditional logistic regression models adjusted for age and sex. Models were further adjusted for known cancer-specific risk factors: lung (BMI, total energy intake, smoking status and pack-years), prostate (BMI, physical activity, smoking status and pack-years), breast (BMI, physical activity, smoking status and pack-years, hormone replacement therapy (HRT)), colorectal (BMI, vegetable consumption, low-dose aspirin use, nonsteroidal anti-inflammatory drugs (NSAID) use, physical activity, smoking status and pack-years), and all cancers (BMI, smoking status and pack-years). BMI (1.3% unknown) and physical activity (total activity MET-hours; 2.4% unknown) were treated as continuous variables, whereas the remaining variables were categorized: smoking status (never, former, current; 0.7% unknown), pack-years (defined as number of packs of cigarettes smoked per day times the number of years the person has smoked; 20 + and < 20 packs/years; 2.8% unknown) vegetable consumption (5 + and < 5 times/day; 0.8% unknown), low-dose aspirin use (2 + and < 2 years; 2.5% unknown), NSAID use (Aspirin, OTC, or Rx; 1 + and < 1 year; 2.5% unknown), and, for women, HRT (ever and never use; 0.6% unknown). Covariate selection was informed by a combination of univariate association analyses in our data (Supplementary Table 1) and/or evidence of established risk factors in the literature. Cases and controls with missing values for the above-mentioned variables were excluded. Sensitivity analyses were conducted to assess the individual associations of regular coffee intake and decaffeinated coffee intake with cancer risk and to test the association with total coffee consumption using only non-drinkers as the reference category (N = 870 among controls).

Statistical significance in two-sided tests was set at P < 0.05, which indicates a 5% probability of a false positive association. All analyses were conducted using the R statistical computing software.

## Supplementary information

Supplementary Tables.

## Data Availability

Data available on request from the authors.

## Abbreviations

SCCS: Southern Community Cohort Study
NSAID: Nonsteroidal anti-inflammatory drugs
HRT: Hormone replacement therapy
