# Supplementary material for: Coffee consumption and cancer risk in African Americans from the Southern Community Cohort Study
Source: Sci Rep. 2020 Oct 21;10:17907. doi: 10.1038/s41598-020-72993-6 (PMC7578784; doi:10.1038/s41598-020-72993-6)
Supplement: Supplementary file 1 — Supplementary Tables. [file 41598_2020_72993_MOESM1_ESM.docx]

**Coffee consumption and cancer risk in African Americans from the Southern Community Cohort Study**

Stephanie L. Schmit^1,2,^*, Onyekachi Nwogu^1^, Marco Matejcic^1^, Amanda DeRenzis^1^, Loren Lipworth^3,4^, William J. Blot^3,5^, Leon Raskin^3^

^1^ Department of Cancer Epidemiology, H. Lee Moffitt Cancer Center and Research Institute, Tampa, FL, USA

^2^ Department of Gastrointestinal Oncology, H. Lee Moffitt Cancer Center and Research Institute, Tampa, FL, USA

^3^ Division of Epidemiology, Department of Medicine, Vanderbilt University Medical Center, Nashville, TN, USA

^4^ Vanderbilt Center for Translational and Clinical Cardiovascular Research, Vanderbilt University Medical Center, Nashville, TN, USA

^5^ The International Epidemiology Institute, Rockville, MD, USA

* schmits3@ccf.org

| **Supplementary Table 1. Characterization of Southern Community Cohort Study African American healthy controls by total coffee consumption** | | | | | | |
| --- | --- | --- | --- | --- | --- | --- |
|  | Controls (n=3,337) | | | | | |
|  | <1 times/day (n=2,229) |  | ≥1 and <2 times/day (n=681) |  | ≥2 times/day (n=375) | p-value^1^ |
| **Age [mean (sd)]** | 54.8 (8.7) |  | 57.7 (9.1) |  | 55.8 (8.7) | 1.13x10^-06^ |
| **Sex [n (%)]^2^** |  |  |  |  |  | 0.142 |
| Male | 1067 (47.9) |  | 346 (50.8) |  | 197 (52.5) |  |
| Female | 1162 (52.1) |  | 335 (49.2) |  | 178 (47.5) |  |
| **Education [n (%)]** |  |  |  |  |  | 0.177 |
| <12 years | 812 (36.4) |  | 276 (40.5) |  | 132 (35.2) |  |
| Graduated high school | 690 (31.0) |  | 196 (28.8) |  | 107 (28.5) |  |
| Some college | 491 (22.0) |  | 154 (22.6) |  | 95 (25.3) |  |
| Graduated college or higher | 234 (10.5) |  | 55 (8.1) |  | 41 (10.9) |  |
| Unknown^3^ | 2 (0.1) |  | 0 (0) |  | 0 (0) |  |
| **Income, US dollar [n (%)]** |  |  |  |  |  | 0.417 |
| <15,000 | 1354 (60.7) |  | 389 (57.1) |  | 233 (62.1) |  |
| 15,000 - 24,999 | 458 (20.5) |  | 143 (21.0) |  | 76 (20.3) |  |
| 24,999 - 49,999 | 275 (12.3) |  | 100 (14.7) |  | 40 (10.7) |  |
| ≥50,000 | 111 (5.0) |  | 38 (5.6) |  | 23 (6.1) |  |
| Unknown | 31 (1.4) |  | 11 (1.6) |  | 3 (0.8) |  |
| **BMI [mean (sd)]** | 30.3 (7.5) |  | 30.3 (6.8) |  | 29.7 (7.2) | 0.237 |
| Total energy intake, kcal/day [mean **(sd)]** | 2522.1 (1481.5) |  | 2695.9 (1465.6) |  | 3108.9 (1585.9) | 2.35x10^-11^ |
| **Total activity MET-hours [mean (sd)]** | 21.5 (19.2) |  | 21.0 (18.2) |  | 22.4 (20.1) | 0.617 |
| **Smoking [n (%)]** |  |  |  |  |  | 3.56x10^-14^ |
| Never | 890 (39.9) |  | 206 (30.2) |  | 90 (24.0) |  |
| Former | 509 (22.8) |  | 191 (28.0) |  | 74 (19.7) |  |
| Current | 818 (36.7) |  | 282 (41.4) |  | 209 (55.7) |  |
| Unknown | 12 (0.5) |  | 2 (0.3) |  | 2 (0.5) |  |
| **HRT [n (%)]^4,5^** |  |  |  |  |  | 0.003 |
| Never (premenopause) | 317 (27.3) |  | 60 (17.9) |  | 37 (20.8) |  |
| Ever (premenopause) | 17 (1.5) |  | 2 (0.6) |  | 3 (1.7) |  |
| Never (postmenopause) | 568 (48.9) |  | 171 (51.0) |  | 88 (49.4) |  |
| Ever (postmenopause) | 256 (22.0) |  | 100 (29.9) |  | 50 (28.1) |  |
| Unknown | 4 (0.3) |  | 2 (0.6) |  | 0 (0) |  |
| **Vegetable consumption, times/day [n (%)]** |  |  |  |  |  | 0.230 |
| <5 | 2183 (97.9) |  | 672 (98.7) |  | 366 (0.976) |  |
| ≥5 | 37 (1.7) |  | 7 (1.0) |  | 9 (0.024) |  |
| Unknown | 9 (0.4) |  | 2 (0.3) |  | 0 (0) |  |
| **Current high cholesterol medication use [n (%)]^6^** |  |  |  |  |  | 0.292 |
| No | 290 (13.0) |  | 111 (16.3) |  | 47 (12.5) |  |
| Yes | 408 (18.3) |  | 147 (21.6) |  | 87 (23.2) |  |
| Unknown | 2 (0) |  | 0 (0) |  | 0 (0) |  |
| **Daily low-dose aspirin use, years [n (%)]** |  |  |  |  |  | 0.022 |
| <2 | 1922 (86.2) |  | 574 (84.3) |  | 305 (81.3) |  |
| ≥2 | 266 (11.9) |  | 95 (14.0) |  | 63 (16.8) |  |
| Unknown | 41 (1.8) |  | 12 (1.8) |  | 7 (1.9) |  |
| **NSAID use, years [n (%)]^7^** |  |  |  |  |  | 0.186 |
| <1 | 1729 (77.6) |  | 519 (76.2) |  | 275 (73.3) |  |
| ≥1 | 464 (20.8) |  | 160 (23.5) |  | 90 (24.0) |  |
| Unknown | 36 (1.6) |  | 2 (0.3) |  | 10 (2.7) |  |
| **Alcohol consumption, times/day [n (%)]^8^** |  |  |  |  |  | 0.384 |
| <1 | 1698 (76.2) |  | 542 (79.6) |  | 293 (78.1) |  |
| ≥1 and <2 | 140 (6.3) |  | 34 (5.0) |  | 19 (5.1) |  |
| ≥2 | 370 (16.6) |  | 99 (14.5) |  | 60 (16.0) |  |
| Unknown | 21 (0.9) |  | 6 (0.9) |  | 3 (0.8) |  |
| **Sweet beverage consumption, times/day [n (%)]^9^** |  |  |  |  |  | 0.068 |
| <1 | 686 (30.8) |  | 207 (30.4) |  | 97 (25.9) |  |
| ≥1 and <2 | 570 (25.6) |  | 174 (25.6) |  | 85 (22.7) |  |
| ≥2 | 962 (43.2) |  | 297 (43.6) |  | 193 (51.5) |  |
| Unknown | 11 (0.5) |  | 3 (0.4) |  | 0 (0) |  |
| **Daily tea intake, times/day [n (%)]** |  |  |  |  |  | < 2.2x10^-16^ |
| <1 | 2053 (92.1) |  | 590 (86.6) |  | 295 (78.7) |  |
| ≥1 and <2 | 108 (4.8) |  | 72 (10.6) |  | 47 (12.5) |  |
| ≥2 | 58 (2.6) |  | 17 (2.5) |  | 33 (8.8) |  |
| Unknown | 10 (0.4) |  | 2 (0.3) |  | 0 (0) |  |
| ^1^Statistical significance for differences between cases and controls was tested using one-way ANOVA for continuous variables and chi-square test for categorical variables | | | | | | |
| ^2^Only males among prostate cancer cases; only females among breast cancer cases | | | |  |  |  |
| ^3^Unknown subjects were excluded from calculations | |  |  |  |  |  |
| ^4^HRT = hormone replacement therapy |  |  |  |  |  |  |
| ^5^In females only |  |  |  |  |  |  |
| ^6^Among those self-reporting high cholesterol |  |  |  |  |  |  |
| ^7^NSAID (nonsteroidal anti-inflammatory drugs) include any of the following drug classes: Aspirin, OTC, Rx; subjects with unknown information for all classes were recoded as unknown in the NSAID use variable | | | | | | |
| ^8^Including beer, wine and liquor |  |  |  |  |  |  |
| ^9^Including soft drinks, Kool-Aid or other sweetened drinks | |  |  |  |  |  |

| **Supplementary Table 2. Association between regular coffee consumption and cancer risk in Southern Community Cohort Study African Americans** | | | | | | |
| --- | --- | --- | --- | --- | --- | --- |
|  |  | Adjusted for age and sex^1^ | |  | Fully adjusted^2^ | |
| Coffee intake (times/day) | n (cases/controls) | OR (95% CI) | p-value^3^ |  | OR (95% CI) | p-value |
| All cancers |  |  |  |  |  |  |
| <1 | 1274/2456 | 1.00 (Ref) |  |  | 1.00 (Ref) |  |
| ≥1 and <2 | 372/614 | 1.15 (1.00-1.33) | 0.050 |  | 1.09 (0.91-1.29) | 0.357 |
| ≥2 | 122/232 | 1.01 (0.80-1.27) | 0.931 |  | 0.93 (0.72-1.22) | 0.620 |
| Lung cancer |  |  |  |  |  |  |
| <1 | 337/2456 | 1.00 (Ref) |  |  | 1.00 (Ref) |  |
| ≥1 and <2 | 127/614 | 1.49 (1.19-1.86) | 5.06x10^-4^ |  | 1.17 (0.90-1.51) | 0.248 |
| ≥2 | 50/232 | 1.58 (1.14-2.19) | 0.006 |  | 0.96 (0.65-1.41) | 0.837 |
| Prostate cancer |  |  |  |  |  |  |
| <1 | 360/1162 | 1.00 (Ref) |  |  | 1.00 (Ref) |  |
| ≥1 and <2 | 104/341 | 0.92 (0.72-1.19) | 0.533 |  | 1.00 (0.74-1.33) | 0.974 |
| ≥2 | 32/114 | 0.88 (0.58-1.33) | 0.538 |  | 0.89 (0.55-1.43) | 0.630 |
| Breast cancer |  |  |  |  |  |  |
| <1 | 346/1294 | 1.00 (Ref) |  |  | 1.00 (Ref) |  |
| ≥1 and <2 | 82/273 | 1.16 (0.88-1.52) | 0.300 |  | 1.12 (0.76-1.65) | 0.565 |
| ≥2 | 18/118 | 0.57 (0.34-0.95) | 0.030 |  | 0.80 (0.43-1.47) | 0.475 |
| Colorectal cancer |  |  |  |  |  |  |
| <1 | 231/2456 | 1.00 (Ref) |  |  | 1.00 (Ref) |  |
| ≥1 and <2 | 59/614 | 1.05 (0.77-1.41) | 0.770 |  | 1.02 (0.61-1.72) | 0.935 |
| ≥2 | 22/232 | 1.02 (0.64-1.61) | 0.940 |  | 0.73 (0.30-1.74) | 0.474 |
| ^1^No sex adjustment for prostate and breast cancers | |  |  |  |  |  |
| ^2^Adjustment factors for: |  |  |  |  |  |  |
| All cancers: age, sex, BMI, smoking status and pack-years | | |  |  |  |  |
| Lung cancer: age, sex, BMI, smoking status and pack-years, total energy intake | | |  |  |  |  |
| Prostate cancer: age, BMI, smoking status and pack-years, physical activity | | |  |  |  |  |
| Breast cancer: age, BMI, smoking status and pack-years, HRT, physical activity | | |  |  |  |  |
| Colorectal cancer: age, sex, BMI, smoking status and pack-years, physical activity, vegetable consumption, low dose aspirin use, NSAID use | | | | | | |
| ^3^p-value from the Wald test |  |  |  |  |  |  |

| **Supplementary Table 3. Association between decaffeinated coffee consumption and cancer risk in Southern Community Cohort Study African Americans** | | | | | | |
| --- | --- | --- | --- | --- | --- | --- |
|  |  | Adjusted for age and sex^1^ | |  | Fully adjusted^2^ | |
| Coffee intake (times/day) | n (cases/controls) | OR (95% CI) | p-value^3^ |  | OR (95% CI) | p-value |
| All cancers |  |  |  |  |  |  |
| <1 | 1590/2982 | 1.00 (Ref) |  |  | 1.00 (Ref) |  |
| ≥1 and <2 | 135/238 | 1.06 (0.85-1.33) | 0.582 |  | 1.04 (0.80-1.36) | 0.722 |
| ≥2 | 43/71 | 1.15 (0.78-1.69) | 0.474 |  | 0.92 (0.58-1.47) | 0.723 |
| Lung cancer |  |  |  |  |  |  |
| <1 | 445/2982 | 1.00 (Ref) |  |  | 1.00 (Ref) |  |
| ≥1 and <2 | 52/238 | 1.50 (1.09-2.06) | 0.013 |  | 1.52 (1.05-2.21) | 0.026 |
| ≥2 | 15/71 | 1.50 (0.85-2.66) | 0.161 |  | 1.00 (0.51-1.96) | 0.991 |
| Prostate cancer |  |  |  |  |  |  |
| <1 | 457/1471 | 1.00 (Ref) |  |  | 1.00 (Ref) |  |
| ≥1 and <2 | 28/115 | 0.73 (0.47-1.12) | 0.145 |  | 0.70 (0.41-1.19) | 0.190 |
| ≥2 | 12/28 | 1.36 (0.68-2.70) | 0.381 |  | 0.86 (0.35-2.14) | 0.747 |
| Breast cancer |  |  |  |  |  |  |
| <1 | 403/1511 | 1.00 (Ref) |  |  | 1.00 (Ref) |  |
| ≥1 and <2 | 32/123 | 1.01 (0.67-1.51) | 0.973 |  | 0.74 (0.41-1.35) | 0.325 |
| ≥2 | 11/43 | 0.96 (0.49-1.89) | 0.915 |  | 0.86 (0.35-2.12) | 0.741 |
| Colorectal cancer |  |  |  |  |  |  |
| <1 | 285/2982 | 1.00 (Ref) |  |  | 1.00 (Ref) |  |
| ≥1 and <2 | 23/238 | 1.01 (0.65-1.58) | 0.955 |  | 0.97 (0.51-1.86) | 0.936 |
| ≥2 | 5/71 | 0.71 (0.29-1.78) | 0.469 |  | 0.50 (0.12-2.09) | 0.400 |
| ^1^No sex adjustment for prostate and breast cancers | |  |  |  |  |  |
| ^2^Adjustment factors for: |  |  |  |  |  |  |
| All cancers: age, sex, BMI, smoking status and pack-years | | |  |  |  |  |
| Lung cancer: age, sex, BMI, smoking status and pack-years, total energy intake | | |  |  |  |  |
| Prostate cancer: age, BMI, smoking status and pack-years, physical activity | | |  |  |  |  |
| Breast cancer: age, BMI, smoking status and pack-years, HRT, physical activity | | |  |  |  |  |
| Colorectal cancer: age, sex, BMI, smoking status and pack-years, physical activity, vegetable consumption, low dose aspirin use, NSAID use | | | | | | |
| ^3^p-value from the Wald test |  |  |  |  |  |  |
